# Supplementary material for: Large-scale rollout of extension training in Bangladesh: Challenges and opportunities for gender-inclusive participation
Source: PLoS One. 2022 Jul 8;17(7):e0270662. doi: 10.1371/journal.pone.0270662 (PMC9269913; doi:10.1371/journal.pone.0270662)
Supplement: Attachment — Submitted filename: PONE-D-21-29046_ReviewComments_ICTEnhancedExtensionTraining.docx [file pone.0270662.s002.docx]

**Review Recommendation**

This manuscript covers an important area of ICT-based extension training in Bangladesh towards gender participation and gender inequalities in women’s access and participation, which has become increasingly important for women's participation in agricultural activities in Bangladesh. Furthermore, the manuscript also explores and compares the gendered impacts from the independent variables and how to increase women’s involvement in ICT-based extension training.

I found this study interesting.

The manuscript is worthy of publication in this journal, but minor amendments listed below need to be addressed.

Nonetheless, my concrete comments are given below by the manuscript section-wise, which are needed to improve the manuscript quality.

**Overall comments:**

a. The manuscript presentation is well enough; however, the sentence structure needs to write in a simple form (especially in the methods section) to be considered a full-length paper and for better readership.
b. Authors should read the whole manuscript several times after correcting the comments below section-wise with the total concentration. And then, the whole manuscript needs to refine/rewrite/reorganize sentences to maintain the sentence consistency and subsequently easy understanding for scientific readership from the preceding sentence to the running sentence.
c. References should be checked for improving with the currently published article.
d. It is not fair not to insert the line numbers in the manuscript; however, authors should insert line numbers in the revised manuscript version.

**Abstract**

1. It is well written, and abstract content has been found systematically organized. However, the sentence before the last sentence needs to improve.

**Introduction**

2. The introduction should be written systematically; it should be organized like current trends of gender participation in ICT-based extension training, gender inequalities in extension training, parameters limitation for women’s participation, variables impacts on women’s participation, review on how to improve women’s assess in participation, research gap, hypothesis, and objectives, etc.

**Methods**

3. On page 5, ‘this was’ should be deleted from the first sentence.
4. There is a blue color sign in Figure 1B? it should be replaced with the new one.
5. Authors should include some valuable pictures and a flow sheet of data collection in the data collection subsection. It gives a clear illumination about the methods section.
6. Authors should cover some theory behind this study and equations to analyze the data.

**Results and Discussion**

Please recheck the results and calculation sheets to delineate the exact and accurate amount to be sure again.

7. For descriptive data paragraph in the results chapter, the structure and meaning of the sentence are proper, but the way of presenting the sentence is not correct need to improve sentence structure more clearly as a simple form for better readership.
8. The discussion is well written.

**Limitations**

9. This section has been written well; however, the authors should mention another limitation- ICT enhanced extension training for empowering women’s skills to be involved more in agricultural works by using the television media.

**Conclusion**

10. The conclusion is well written in a descriptive way, not written in numerative and contains recommendations.
