# Supplementary material for: Large-scale rollout of extension training in Bangladesh: Challenges and opportunities for gender-inclusive participation
Source: PLoS One. 2022 Jul 8;17(7):e0270662. doi: 10.1371/journal.pone.0270662 (PMC9269913; doi:10.1371/journal.pone.0270662)
Supplement: Attachment — Submitted filename: PONE-D-21-29046_review.docx [file pone.0270662.s003.docx]

**PONE-D-21-29046: Large-scale rollout of information and communication technology-enhanced extension training in Bangladesh demonstrates challenges and opportunities towards inclusive gender participation**

The study provides critical insights into where and when Bangladeshi agricultural communities are more likely to access and participate in extension training services. Moreover, it also demonstrates that ICT-enhanced educational programs have both the potential for scaling to large numbers of people and the potential to amplify gender disparities in terms of female participation. The study satisfied almost all the criteria to publish as an original research paper.

However, handing the followings would improve its quality:

1. I would like to suggest the authors to simplify the title.
2. In the abstract, please write eight administrative divisions, not division.
3. At the end of page#4, *For instance, educational ICT…….attended events* requires reference. Also, for the other lines of the paragraph, at least two more references are required.
4. Methods: *This was* mentioned two times (please check page#5)
5. In page#6, Majumder, 2013 is very old information for rice crop. Please, check latest studies done by BRRI researcher for updated information on rice.
6. In the data collection procedure, please mention the number enumerators per session and the time spent per respondent while interview.
7. Data analysis: Why binomial regression with logit? Why not other models? such as OLS, MLE, Frictional logit, probit, etc. Please, justify the utilization of statistical tools.
8. Descriptive data: *10 sessions were missing information* should be *10 sessions had missing information.*
9. In page#9 paragraph#2, your finding described that if the trainer is male, the total participation increased by 16%, but female participants reduced by 47%. This is interesting, but did the participants new about the gender of the trainer before the training session? In general, the participants do not know before the session start about the trainer and do not impact on the number of participants.
10. You also find out that women are more likely to attend in the training on Tuesday. But, why Tuesday? Why less likely to other days? Explaining the reasons in the discussion would generate important policy guidelines.
11. Also, why women likely to attend in January than October? Please, explain the reasons in the discussion.
12. What I was expecting at the end of this paper, the policy options for enhancing agricultural extension (most importantly for the development of women friendly agricultural extension policy) is lacking in this study. I would like to request the authors to review the agricultural extension policy of Bangladesh and suggest to fill up the gaps in the light of findings of this study.
