# Supplementary material for: Large-scale rollout of extension training in Bangladesh: Challenges and opportunities for gender-inclusive participation
Source: PLoS One. 2022 Jul 8;17(7):e0270662. doi: 10.1371/journal.pone.0270662 (PMC9269913; doi:10.1371/journal.pone.0270662)
Supplement: Attachment — Submitted filename: PONE-D-21-29046_ReviewComments.docx [file pone.0270662.s004.docx]

**PlosOne**

**Point-by-Point Response to the Editor and Reviewers**

*Dear editor and reviewers,*

*We thank you for your your thoughtful comments. We have made changes to the paper based on your comments (see the point-by-point response below). Our comments are in italics. Parts taken from the paper are in quotation marks (“ ”), and new sections added are underlined.*

**Previous Title:** Large-scale roll-out of information and communication technologies extension training in Bangladesh demonstrates challenges and opportunities towards inclusive gender participation

**Revised Title**: ***Large-scale rollout of extension training in Bangladesh: Challenges and opportunities for gender-inclusive participation***

**Authors:** John William Medendorp, N. Peter Reeves, Victor Giancarlo Sal y Rosas Celi, Md. Harun-ar-Rashid, Timothy J. Krupnik, Anne Lutomia, Barry Pittendrigh, and Julia Bello-Bravo.

**Editor Comments**

**An useful paper with some interesting findings. I agree with the observations made by two reviewers and suggest authors revise the paper accordingly. Some of my observations are as follows:**

*Thank you for your positive feedback.*

1. **Please follow the PLOS one guideline to arrange the different sections of the paper. For example, the limitation should be part of the discussion NOT as part of results.**

*We have modified the paper to be compliant with PLOS One guidelines to which you referred us, including statistical reporting requirements for observational studies (STROBE).*

2. **Some of the details provided as part of descriptive analysis should actually be part of method section. Similarly, the data section instead of telling about data, it details the program. I suggest renaming the section as intervention and include a new section data where describe how the data was gathered and processed? How was the quality control done?**

*We have moved the descriptive analysis section to the methods section. We have also renamed the section, intervention section, and added a new section describing how the data was collected.*

*In terms of quality control, we have added the following sentences:*

*“As described above, data was gathered based on registered participants in training sessions. Therefore, non-prior specification of data management was implemented.”*

3. **Justify the use of statistical methods, specifically negative binomial.**

*We have rewritten this section to read as follows:*

*“In order to assess factors associated with the expected number of persons attending a given event, a negative binomial regression model using a log link was fitted. Negative binomial, instead of Poisson model, was fitted because the variance of the dependent variable was greater than the mean.*

*The odds of females attending the event, given the total number of persons attending, was assessed using a binomial regression model with a logit link, where the total number of females (number of successes) and the total number of participants (number of trials) were the composite outcome variable.”*

4. **For timing of the authors switched from 4 category to 3 category and justified it through statistical test. I am wondering if authors did for other categorical variables. If yes, please include the test results as supplementary files and write about in the analysis section.**

*We did not assess the need to reduce the number of categories for other variables.*

5. **The English language needs a thorough review.**

*We have made significant changes to the manuscript to improve readability, including sentence structure, elimination of ambiguities, and elimination of redundencies, based on the comments of the editor and reviewers.*

**Review #1 Comments**

This manuscript covers an important area of ICT-based extension training in Bangladesh towards gender participation and gender inequalities in women’s access and participation, which has become increasingly important for women's participation in agricultural activities in Bangladesh. Furthermore, the manuscript also explores and compares the gendered impacts from the independent variables and how to increase women’s involvement in ICT-based extension training. I found this study interesting.

*Thank you for your positive feedback.*

**Overall comments:**

6. **The manuscript presentation is well enough; however, the sentence structure needs to write in a simple form (especially in the methods section) to be considered a full-length paper and for better readership.**

**Authors should read the whole manuscript several times after correcting the comments below section-wise with the total concentration. And then, the whole manuscript needs to refine/rewrite/reorganize sentences to maintain the sentence consistency and subsequently easy understanding for scientific readership from the preceding sentence to the running sentence.**

*Thank you for this suggestion. As you will see from the mark-up copy that we have included with our submission, we have made significant changes to the manuscript to improve readability, including sentence structure, based on the comments of the editor and reviewers.*

7. **References should be checked for improving with the currently published article.**

*All references were checked for accuracy.*

8. **It is not fair not to insert the line numbers in the manuscript; however, authors should insert line numbers in the revised manuscript version.**

*We apologize for not including line numbers in the submitted manuscript. We have added line numbers in the mark-up version of the resubmitted manuscript.*

**Abstract**

9. **It is well written, and abstract content has been found systematically organized. However, the sentence before the last sentence needs to improve.**

*Thank you for your comment. We have modified the last sentence as follows:*

*"* *The study concludes with a discussion of ways to mitigate gender exclusion in extension training by improving future applications “big data” analyses improve women’s participation in extension services."*

**Introduction**

10. **The introduction should be written systematically; it should be organized like current trends of gender participation in ICT-based extension training, gender inequalities in extension training, parameters limitation for women’s participation, variables impacts on women’s participation, review on how to improve women’s assess in participation, research gap, hypothesis, and objectives, etc.**

*Gender inequalities in extension training, parameters for women’s participation, variables impacting women’s participation are included in the discussion section so we did not include them here. How to improve women’s access and participation is part of the conclusions of the paper, so we have not included them here.*

*Since this is an observational study, and not an experimental nor quasi-experimental design, we have not included a hypothesis but have made more explicit our objective and research question by including the following text:*

*“**Our objective for this study is to analyze the effects of situational factors – time of day, gender of trainer, day of the week, month of the year, Administrative District, and venue type – on participation in extension events in general, and women’s participation in particular. Our research questions therefore are: 1) what factors increased women’s participation in ICT-based extension events and, 2) What are the odds of women attending any given event?”*

*In response to the reviewer’s recommendations, we have made reference to discussion section in the introduction so that the reader knows that these topics are discussed in a later section of the paper.*

**Methods**

11. **On page 5, ‘this was’ should be deleted from the first sentence.**

*We have rewritten this sentence to read:*

*“The research design for this paper is a convenience-sampled, retrospective observational study based on the analysis of data collected through extension events at selected venues throughout Bangladesh.”*

12. **There is a blue color sign in Figure 1B? it should be replaced with the new one.**

*We have replaced Figure 1B.*

13. **Authors should include some valuable pictures and a flow sheet of data collection in the data collection subsection. It gives a clear illumination about the methods section.**

*We have inserted a graphic to depict the data collection and data analysis process.*

14. **Authors should cover some theory behind this study and equations to analyze the data.**

*We have added the following description to the methods section covering the theory for the models.*

*“In order to assess factors associated with the expected number of persons attending a given event, a negative binomial regression model using a log link was fitted. Negative binomial, instead of Poisson model, was fitted because the variance of the dependent variable was greater than the mean.*

*The odds of females attending the event, given the total number of persons attending, was assessed using a binomial regression model with a logit link, where the total number of females (number of successes) and the total number of participants (number of trials) were the composite outcome variable.”*

**Results and Discussion**

15. **Please recheck the results and calculation sheets to delineate the exact and accurate amount to be sure again.**

*We checked the results, including numbers in the tables and they are correct.*

16. **For descriptive data paragraph in the results chapter, the structure and meaning of the sentence are proper, but the way of presenting the sentence is not correct need to improve sentence structure more clearly as a simple form for better readership.**

*We modified the descriptive data paragraph to be clearer as follows:*

*“The mean number of persons attending a given session was 123 (SD=103.7), and the mean number of females attending a given session was 23 (SD=25.8). The most common number of persons attending training was 41 (101 events representing 9.4% of total sessions), and for females it was zero (254 events representing 23.7% of total sessions) (Figure 2). In terms of trainers, only 104 (9.7%) were conducted by female extension agents. Most sessions were conducted in Dinajpur (n=529, 49.4%), followed by Jashore (n=295, 27.6%) and Faridpur (n=202, 19.0%), with the least being Rajshahi (n=42, 4.0%). Sessions were distributed relatively evenly among the days of the week and occurred most frequently in the month of December.”*

17. **The discussion is well written.**

*Thank you for the positive feedback.*

**Limitations**

18. **This section has been written well; however, the authors should mention another limitation- ICT enhanced extension training for empowering women’s skills to be involved more in agricultural works by using the television media.**

*We included a statement in the limitation section as follows:*

*"There were several limitations with the study that should be considered. First, this was a convenience-sampled, retrospective observational study. Second, only limited information associated with session attendance instead of individual participant details were available and therefore underlying factors such as age, education level, religious affiliation, and socio-economic status, among others, of the persons attending the session could not be assessed. Furthermore, our models assume that the attending (or not) of an individual person is conditionally independent of other unobserved factors. Additionally, not all Bangladesh Divisions were represented in the study. This was a result of the nature of the training sessions administered: rather than focus on covering the whole country, they were concentrated into project areas in which maize, rice, and wheat are intensively grown. However, given that half of the divisions in Bangladesh were included, and that there was a large geographical coverage within each Division, the date collected still provide opportunity to explore potential differences for female training participation.* *Finally, it is important to not that the results should be viewed as specific to this particular type of training approach. In the future, other forms of information disemination (e.g. social media and television campaigns) could also be studied to determine if gender specific preferences exists."*

**Conclusion**

19. **The conclusion is well written in a descriptive way, not written in numerative and contains recommendations.**

*Thank you for the positive feedback.*

**Review #2 Comments**

20. **The study provides critical insights into where and when Bangladeshi agricultural communities are more likely to access and participate in extension training services. Moreover, it also demonstrates that ICT-enhanced educational programs have both the potential for scaling to large numbers of people and the potential to amplify gender disparities in terms of female participation. The study satisfied almost all the criteria to publish as an original research paper.**

*Thank you for the positive feedback.*

21. **I would like to suggest the authors to simplify the title.**

*Based on the reviewer's suggestion we simplified the title name to:*

"*Large-scale rollout of extension training in Bangladesh: Challenges and opportunities for gender-inclusive participation”*

22. **In the abstract, please write eight administrative divisions, not division.**

*"Administrative Division" was changed to "Administrative Divisions".*

23. **At the end of page#4, *For instance, educational ICT…….attended events* requires reference. Also, for the other lines of the paragraph, at least two more references are required.**

*We have added references to support this claim.*

24. **Methods: *This was* mentioned two times (please check page#5)**

*We have rewritten this sentence to read:*

*“The research design for this paper is a convenience-sampled, retrospective observational study based on the analysis of data collected through extension events at selected venues throughout Bangladesh.”*

25. **In page#6, Majumder, 2013 is very old information for rice crop. Please, check latest studies done by BRRI researcher for updated information on rice.**

*Updated references were provided.*

*"* *rice is a primary staple food that provides 48% of rural employment, about two-thirds of the overall supply of calories, and about half of the average person's total protein intake within the country, occupying around 74% of the total cropped area, which accounts for 50% of Bangladesh's agricultural GDP and a sixth of its national income”*

*“Chakma, K., & Ruba, U. B. (2021). Role of Bangladeshi Women in Diverse Agricultural Production: A Review. European Journal of Agriculture and Food Sciences, 3(3), 1-5. doi:10.24018/ejfood.2021.3.3.284.”*

*“Bangladesh Bureau of Statistics. (2018). 45 years of agriculture statistics of major crops. Statistics and Informatics Division (SID), Ministry of Planning, Government of the People's Republic of Bangladesh.”*

26. **In the data collection procedure, please mention the number enumerators per session and the time spent per respondent while interview.**

*The number of enumerators per session and time spent were added.*

*"* *At each event, three trained enumerators collected data . . . "* [line 119]

27. **Data analysis: Why binomial regression with logit? Why not other models? such as OLS, MLE, Frictional logit, probit, etc. Please, justify the utilization of statistical tools.**

*Justification for the use of the statistical tools was provided.*

*“In order to assess factors associated with the expected number of persons attending a given event, a negative binomial regression model using a log link was fitted. Negative binomial, instead of Poisson model, was fitted because the variance of the dependent variable was greater than the mean.*

*The odds of females attending the event, given the total number of persons attending, was assessed using a binomial regression model with a logit link, where the total number of females (number of successes) and the total number of participants (number of trials) were the composite outcome variable.”*

28. **Descriptive data: *10 sessions were missing information* should be *10 sessions had missing information.***

*The sentence was changed.*

*“Among the 1,080 sessions conducted, 10 sessions had missing information on the gender of the trainer; therefore, only 1,070 sessions were included in the analysis.”*

29. **In page#9 paragraph#2, your finding described that if the trainer is male, the total participation increased by 16%, but female participants reduced by 47%. This is interesting, but did the participants new about the gender of the trainer before the training session? In general, the participants do not know before the session start about the trainer and do not impact on the number of participants.**

*We have added text to indicate that women would not have known the gender of the trainer before arriving at the venue. We are not certain whether this factored into their decision to remain or not:*

*“However, since in our study attendees would not have known the gender of the trainer until arriving at the event, further research is needed in order to understand whether this was a factor in the decision to stay or leave.”*

30. **You also find out that women are more likely to attend in the training on Tuesday. But, why Tuesday? Why less likely to other days? Explaining the reasons in the discussion would generate important policy guidelines.**

*We added text to indicate that we do not have an explanation for this finding at this time:*

*“There is a clear preference for Tuesdays for women’s participation in our results. We do not at this time have an explanation for this result. This would require further research.”*

*31.* **Also, why women likely to attend in January than October? Please, explain the reasons in the discussion.**

*We added text to indicate that we do not have an explanation for this finding at this time:*

*“There is a clear preference in our results for Tuesdays for women’s participation. We do not at this time have an explanation for this result. This would require further research. In addition, there was a clear trend toward greater participation of women in the later months of the program than for the earlier months, with January being the month of highest participation. We are not certain whether this result was due to learning on the part of the program implementers to improve their outreach to women, or whether seasonal factors may have played a role. In the agricultural cycle in Bangladesh, January is the month of least agricultural activity. This may have been a factor in the increased participation, but would require further research to confirm.”*

31. **What I was expecting at the end of this paper, the policy options for enhancing agricultural extension (most importantly for the development of women friendly agricultural extension policy) is lacking in this study.** **“I would like to request the authors to review the agricultural extension policy of Bangladesh and suggest to fill up the gaps in the light of findings of this study.”**

*Based on the reviewer comments, we added specific recommendations to the conclusion and added some text as part of a third recommendation.*

***Make female participation in extension training a priority***

*“Finally, in order to enhance development outcomes, maximizing female participation in extension trainings should be a priority. A recent study (Chackma et al., 2021) showed that females participate in most components of rice production in Bangladesh, although their activities tended to be mostly concentrated in the area of post-harvest (cleaning, drying, storing, bagging, income management, and sorting). Although female participation undoubtedly varies from crop to crop, it is safe to assume that it is significant across the commodity groups. This alone should warrant specific strategies to provide extension services that are developed specifically for female participants in the production value chain, either jointly with males, or separately to address those parts of the value chain that a supported mostly by females. Only when extension services are truly inclusive will it be possible to maximize agricultural potential.”*

*Thank you again to the editor and the reviewers for your constructive comments. We hope that we have addressed your concerns adequately.*
