# Supplementary material for: Large-scale rollout of extension training in Bangladesh: Challenges and opportunities for gender-inclusive participation
Source: PLoS One. 2022 Jul 8;17(7):e0270662. doi: 10.1371/journal.pone.0270662 (PMC9269913; doi:10.1371/journal.pone.0270662)
Supplement: Attachment — Submitted filename: PONE-D-21-29046R1_comments.docx [file pone.0270662.s005.docx]

**Manuscript Number:** PONE-D-21-29046R1
**Manuscript Title:** Large-scale rollout of extension training in Bangladesh: Challenges and opportunities for gender-inclusive participation

Comments on the revised manuscript

1. I found the authors have addressed all the comments and tried to fix them up. However, I am not fully satisfied with the response to comments# 29, 30, and 31. The authors did not get information from their survey to meet these comments and demanded further study/investigation required for that. Mentioning the limitations of the study and leaving these for further investigation is scientific. However, I would like to request to search in the literature if the author can find any supporting information to support/explain these findings.
2. I also would like to make the policy implication more gorgeous. The study has enough resources to critique the national agricultural extension policy (NAEP) 2020 (in Bengali). Whether the NAEP emphasizes training the women participants or not. How about the ICT-based training? In the social aspect, do the NAEP realizes the importance of female trainer for enhancing female participants in the training program? If the authors find these issues addressed in the NAEP, can mention them in the justification of policy implication section. If these are found gaps in the NAEP, can suggest to include in the implementation guideline of NAEP.

Finally, I found the paper improved significantly and the editor may accept it with minor revisions.
