# Supplementary material for: Large-scale rollout of extension training in Bangladesh: Challenges and opportunities for gender-inclusive participation
Source: PLoS One. 2022 Jul 8;17(7):e0270662. doi: 10.1371/journal.pone.0270662 (PMC9269913; doi:10.1371/journal.pone.0270662)
Supplement: Attachment — Submitted filename: PONE-D-21-29046R1_ReviewComment_LargeScaleRoleout.docx [file pone.0270662.s006.docx]

**Review Recommendation**

I am pleased that the authors are responded to all the comments. The response to the comments provided by the authors is sufficient which could be considered to publish this manuscript and the response to the comments for the section of the introduction, materials and methods, results, discussion, conclusion and reference is, however, consistently organized and well written.

Remember that when proofreading will be performed, everything should be corrected.
