# Supplementary material for: Large-scale rollout of extension training in Bangladesh: Challenges and opportunities for gender-inclusive participation
Source: PLoS One. 2022 Jul 8;17(7):e0270662. doi: 10.1371/journal.pone.0270662 (PMC9269913; doi:10.1371/journal.pone.0270662)
Supplement: Attachment — Submitted filename: Large-scale rollout Response to Reviewers Version 3.docx [file pone.0270662.s007.docx]

**PlosOne**

**Point-by-Point Response to Reviewers**

*Dear editor and reviewers,*

*We thank you for your thoughtful comments. We have made changes to the paper based on your comments (see the point-by-point response below). Our comments are in italics. Parts taken from the paper are in quotation marks (“ ”), and new sections added are underlined.*

Responses to Reviewers Comments

**Reviewer #1:**

**I am pleased that the authors are responded to all the comments. The response to the comments provided by the authors is sufficient which could be considered to publish this manuscript and the response to the comments for the section of the introduction, materials and methods, results, discussion, conclusion and reference is, however, consistently organized and well written.
Remember that when proofreading will be performed, everything should be corrected.**

*Thank you for your review. We are grateful for your constructive engagement with the editing process.*

**Reviewer #2: Comments on the revised manuscript**

**1. I found the authors have addressed all the comments and tried to fix them up. However, I am not fully satisfied with the response to comments# 29, 30, and 31. The authors did not get information from their survey to meet these comments and demanded further study/investigation required for that. Mentioning the limitations of the study and leaving these for further investigation is scientific. However, I would like to request to search in the literature if the author can find any supporting information to support/explain these findings.**

*Thank you for that request. We realized upon reviewing the uploaded documents, that we had not uploaded the correct version of the paper. We are including our responses to the previous version as well as these new comments so that you can see how the paper has changed.*

**29. In page#9 paragraph#2, your finding described that if the trainer is male, the total participation increased by 16%, but female participants reduced by 47%. This is interesting, but did the participants new about the gender of the trainer before the training session? In general, the participants do not know before the session start about the trainer and do not impact on the number of participants.**

*We have conducted a thorough review of the relevant literature as requested by the reviewer. In addition, we have engaged extensively with colleagues in Bangladesh in order to discern some of the possible answers to the reviewer’s questions and concerns. We devote a significant portion of the discussion section addressing the literature on women’s preference for female-led events. We have amended the text accordingly:*

*"**Our results also support other findings that women participants tended to be more comfortable in settings led by other women than by men [45-46, 44], and that female-led training increases women’s participation [45- 50]. The increased presence of women agents in extension systems is something that has long been advocated and our results show that the presence of women agents clearly makes a statistical difference in women’s participation. However, since in our study attendees may not have known the gender of the trainer until arriving at the event, further research is needed in order to understand whether this was a factor in the decision to stay or leave."*

**30. You also find out that women are more likely to attend in the training on Tuesday. But, why Tuesday? Why less likely to other days? Explaining the reasons in the discussion would generate important policy guidelines.**

*Unfortunately, the study was not designed to address why preferences in days exists and there is no clear insights from the literature. So any explanations for women training preferences on Tuesday (and Wednesday) would be speculation. However, at the request of the reviewer, we have added some additional discussion on this topic in the text.*

*“**In this regard, two of our results remain unexplained. Our data suggests that both Tuesday and Wednesday tend to increase female participation more than the other days and that there is clear evidence to suggest that both Sunday and Thursday decrease female participation. The reasons for these female preferences is unclear but could be related to Sunday being the first day in the work week and Thursday being the last and that these two days are less convenient for females. However, further research is needed to understand the impact that day of the week has in the likelihood of females participating in training. There is a substantial body of research on women’s time allocation in rural Bangladesh [51, 52]; however, these studies address only the allocation of time in the aggregate but do not discuss the question of day of the week time allocation. This is an area that would merit additional research.”*

*We have also added two new references:*

*“51. Khandker, S. R. (1988). Determinants of women's time allocation in rural Bangladesh. Economic Development and Cultural Change, 37(1), 111-126.*

*52. Islam, F. B., & Sharma, M. (2022). Socio-economic determinants of women’s livelihood time use in rural Bangladesh. GeoJournal, 1-13.”*

*31.* **Also, why women likely to attend in January than October? Please, explain the reasons in the discussion.**

*We added text to indicate that we do not have an explanation for this finding at this time:*

*“In the agricultural cycle in Bangladesh, January comes at the end of the Aman growing cycle which is the time of greatest crop production in Bangladesh, with rice being the principle crop [53-54]. According to colleagues, this is a time of year when many of those involved have more leisure, depending, of course, when they choose to plant their winter crops.”*

We have also added two new references:

*“53. Sultana, S., Khan, M. A., Hossain, M. E., Prodhan, M. M. H., & Saha, S. M. (2022). Yield gap, risk attitude, and poverty status of aman rice producers in climate-vulnerable coastal areas of Bangladesh. Journal of Agricultural Science and Technology, 0-0.*

*54. Al Mamun, M. A., Nihad, S. A. I., Sarkar, M. A. R., Aziz, M. A., Qayum, M. A., Ahmed, R., ... & Kabir, M. S. (2021). Growth and trend analysis of area, production and yield of rice: A scenario of rice security in Bangladesh. PloS one, 16(12), e0261128.”*

**2. I also would like to make the policy implication more gorgeous. The study has enough resources to critique the national agricultural extension policy (NAEP) 2020 (in Bengali). Whether the NAEP emphasizes training the women participants or not. How about the ICT-based training? In the social aspect, do the NAEP realizes the importance of female trainer for enhancing female participants in the training program? If the authors find these issues addressed in the NAEP, can mention them in the justification of policy implication section. If these are found gaps in the NAEP, can suggest to include in the implementation guideline of NAEP.**

*We have reviewed the National Agricultural Extension Policy and have incorporated citations and added the following text to support our recommendations:*

“*The recent agricultural extension policy published by the Bangladesh Ministry of Agriculture [69] highlights both gender issues and e-extension efforts as part of their priorities. The inclusion of women is mentioned seven times in the document (**§§ 1.3, 1.4, 3.2, 4.1, 4.3, and 5). Especially the last of these references speaks to the Ministry’s commitment to “taking appropriate expansion measures to increase the participation of women at all levels of modern agricultural production and marketing system” (p. 14). Likewise, support for e-agriculture is an important part of the Ministry’s strategy (§3.2).”*

*“The Bangladesh National Agricultural Extension Policy [69] already commits itself to automatic data collection on climate as part of its strategy (§3.2). A similar commitment should be made to collecting information on women’s participation for the purpose of improving women’s access to agricultural information.”*

*“This should include specific steps to increase the number of women extension agents in order to increase the odds of women’s participation in extension training events like those described in this study.*”

**Finally, I found the paper improved significantly and the editor may accept it with minor revisions.**

*Thank you for your kind comments.*
